# Subacromial impingement in patients with whiplash injury to the cervical spine

**DOI:** 10.1186/1749-799X-3-25

**Published:** 2008-06-27

**Authors:** Ali Abbassian, Grey E Giddins

**Affiliations:** 1 Specialist Registrar in Trauma & Orthopaedics, North-West Thames Training programme, Mayday University Hospital, 530 London Road, Croydon, CR7 7YE, UK; 2 Consultant Orthopaedic and Hand Surgeon, Hand to Elbow Clinic, 29A James street west, Bath, UK

## Abstract

**Background:**

Impingement syndrome and shoulder pain have been reported to occur in a proportion of patients following whiplash injuries to the neck. In this study we aim to examine these findings to establish the association between subacromial impingement and whiplash injuries to the cervical spine.

**Methods and results:**

We examined 220 patients who had presented to the senior author for a medico-legal report following a whiplash injury to the neck. All patients were assessed for clinical evidence of subacromial impingement. 56/220 patients (26%) had developed shoulder pain following the injury; of these, 11/220 (5%) had clinical evidence of impingement syndrome. Only 3/11 patients (27%) had the diagnosis made prior to evaluation for their medico-legal report. In the majority, other clinicians had overlooked the diagnosis. The seatbelt shoulder was involved in 83% of cases (p < 0.03).

**Conclusion:**

After a neck injury a significant proportion of patients present with shoulder pain, some of whom have treatable shoulder pathology such as impingement syndrome. The diagnosis is, however, frequently overlooked and shoulder pain is attributed to pain radiating from the neck resulting in long delays before treatment. It is important that this is appreciated and patients are specifically examined for signs of subacromial impingement after whiplash injuries to the neck. Direct seatbelt trauma to the shoulder is one possible explanation for its aetiology.

## Background

Whiplash injuries of the cervical spine are common. Additionally these injuries have a high incidence of legal action and employment loss. There are a number of well documented symptoms associated with whiplash injuries. These may include neck pain, occipital headaches, thoraco-lumbar back pain, parasthesia, weakness, visual disturbances, vertigo and even dysphagia [1-4]. Pain radiating to the upper limbs and/or shoulders is a common symptom. Additionally shoulder and neck pain can often co-exist and the differentiation of cervical radiculitis from primary shoulder disease at times can be difficult [5].

Impingement syndrome, as a separate entity, however, has less established links with neck injuries. Chauhan and colleagues examined 524 patients who presented to the Accident and Emergency department and reported a 9% incidence of impingement type pain [6]. It has even been suggested that subacromial impingement can present as an asymptomatic variant and with neck pain alone [7].

In this paper we review the incidence of impingement syndrome in association with whiplash injuries in a group of patients presenting for medicolegal claims and review the relevant literature.

## Patients and Methods

Individuals presenting to the senior author in a 10-year period for a medico-legal report who had suffered a whiplash injury, were assessed prospectively for evidence of subacromial impingement. Whiplash was considered when the individual was complaining of pain and aching to the neck in the presence or absence of restriction of neck movements secondary to a hyper flexion/extension injury caused by their recent accident. Those with neck or shoulder symptoms prior to the index injury were excluded from the study.

Anyone with shoulder pain was evaluated for clinical evidence of impingement syndrome. This involved a full examination of the neck and shoulder and assessing for evidence of subacromial impingement. The diagnosis was made on the basis of the following clinical tests: the Neer impingement sign [8], Hawkins-Kennedy impingement sign [9] the painful arc sign and supraspinatus muscle strength test. Records were made of the details of any radiological imaging that was performed before as well as after the medicolegal report. When appropriate, radiographs of the neck and shoulder had been taken to rule out any bony injury. Further imaging (as part of the medicolegal assessment) was not, however, routinely obtained.

The inclusion criteria were therefore anyone with a new onset shoulder pain following their neck injury as well as having four positive clinical tests as described above.

If the injury was sustained in a Motor Vehicle Accident (MVA) the position of the patient in the car, site of impact and the use of headrests and seatbelts were documented.

## Results

220 medico-legal reports were reviewed retrospectively. Patients had been examined an average of 13.4 months (range 1–59 months) following their accident. Male to female ratio was 1:1.3 with an average age of 38 years (range 10–83 years).

202/220 of the patients (92%) were involved in an MVA. 129/220 (64%) were as a result of rear impact. The remaining 18 patients were riding motorbikes or bicycles, or were pedestrians. 161/202 of the car accident victims (80%) were drivers and 36/202 (18%) front seat passengers. Only 3/202 (1.5%) individuals were not wearing a seatbelt and 5/202 (2.5%) did not have a headrest in position at the time of the accident.

Although none had an associated cervical spine fracture, 9/220 patients (4%) had sustained fractures of the limbs or the skull. 133/220 patients (60%) had a concomitant soft tissue injury to their thoracic or lumbar spine and had complained of back pain after the incident. 21/220 patients (9.5%) had also sustained a minor head injury at the time of the accident.

A total of 56/220 patients (26%) had shoulder pain following the injury, of these 11/220 (5%) had signs and symptoms consistent with subacromial impingement (Table 1). In the other 45 patients the symptoms were radiation from the neck and no clinical or radiological evidence of primary shoulder pathology was identified. All 11 patients with evidence of subacromial impingement were involved in car accidents and 9/11 (81%) of them were drivers. In one patient both shoulders were involved and thus 12 shoulders with clinical evidence of impingement syndrome were identified. The seatbelt shoulder (driver's right and front passengers' left – all were right hand drive cars) was implicated in 10/12 shoulders (83%) (X^2^, P = 0.021). In the 2 shoulders that the non-seatbelt side was involved there had been documentation of direct injury to the non-seatbelt side of the body in the patients' medical notes at presentation. All of the patients had noticed pain in their affected shoulder within the first week after the injury and none had pre-existing shoulder symptoms.

**Table 1:** Patients with subacromial impingement following neck injuy

**Patient**	**Sex**	**Age**	**Side**	**Position**	**Pain first noted**	**Seen by before diagnosed**	**Diagnosed by**	**Months to diagnosis**	**Mode of Diagnosis**
1	F	68	Right	Driver	Day 1	GP, Physio	Specialist	16	MRI/Clinical
2	M	60	Right	Driver	Day 2	GP	Report	4	US/Clinical
3	M	60	Right	Driver	Day 7	GP, Physio	Physio	20	Clinical
4	M	66	Left	Driver	Day 4	GP	Report	5	Clinical
5	M	49	Right	Driver	Day 1	GP	Report	6	US/Clinical
6	M	55	Right	Driver	Day 6	GP, Physio, Chiropractor	Report	5	Clinical
7	M	47	Right	Driver	Day 2	GP	Report	6	MRI/Clinical
8	F	69	Right	Driver	Day 4	GP, Physio	Report	3	Clinical
9	M	84	Bilateral	Driver	Day 1	Inpatient, GP	Specialist	3	MRI/Clinical
10	F	18	Left	Front passenger	Day 1	GP	Report	5	US/Clinical
11	F	68	Left	Front passenger	Day 1	GP	Report	1	Clinical

All patients had been seen by their general practitioner but only one had been referred for specialist treatment. 3/11 (27%) patients had had their subacromial impingement diagnosed prior to the medicolegal report (table 1). From the three patients who were diagnosed prior to the report, only one was diagnosed in the primary care sector, by a physiotherapist who was delivering the 'neck' therapy. The remainder had their diagnosis made at the time of our report and subsequently advised to seek further medial assessment. Mean time to diagnosis was 8.8 months (range 2–20).

The group of patients who developed subacromial impingement were on average older than the patients who did not. 57.5 years verses 36.9 years (t-test, p = 0.002).

## Discussion

The incidence of shoulder pain following soft-tissue injuries to the neck is variable. In a prospective study of 93 car-accident victims, 16 (18%) were found to have shoulder symptoms at follow-up [10]. Others have quoted higher figures but it is not clear what proportion, if any, had impingement syndrome as a specific diagnosis. Chauhan and colleagues examined 102 patients for evidence of impingement syndrome [6]. The incidence of shoulder pain was found to be 22% but only 9% had subacromial impingement. Following soft tissue injuries to the neck up to a third of the patients can be expected to develop shoulder pain. The incidence of subacromial impingement however is less well established. In our series 26% of patients had developed shoulder symptoms, which is comparable to figures quoted above, but only 5% were found to have clinical signs of impingement syndrome on an average of 13 months after injury.

All our patients were involved in litigation and may therefore have different characteristics. It has been shown that long-term disability following neck injury is unrelated to the physical insult and those pursuing compensation have the highest physical disability in terms of neck pain [11]. Although this has not been specifically validated for impingement syndrome following neck injuries, a similar outcome can be expected.

In our review of the literature we identified two other studies that reported shoulder pain and subacromial impingement following whiplash injuries to the neck [6,12]. Gorski [7] described asymptomatic impingement syndrome: where patients with neck pain alone responded to subacromial injections with a complete or a substantial relief of their neck pain. They postulated that chronic neck pain can be caused by subacromial impingement which should be considered in the differential diagnoses even if the shoulder is asymptomatic.

In our study clinical examination was the main tool for diagnosing subacromial impingement although some of our patients (table 1) did have radiological confirmation. Clinical tests in combination have been shown to have high post test probabilities for rotator cuff pathology [13]. Muddu et al [12] have suggested that the primary pathology is due to a whiplash injury to the shoulder, as a separate entity, rather than impingement syndrome. In their series 15 out of 18 patients who were found to have 'shoulder symptoms' by a consultant orthopaedic surgeon had no significant shoulder pathology on MRI. In fact only 2 from 18 patients (11%) demonstrated rotator cuff tears and evidence of subacromial impingement. It is not clear however if their patients had positive clinical signs for subacromial impingement (despite their negative MRI) or they were merely complaining of generalised shoulder pain following their neck injury.

Pain radiating from the neck to the shoulder after whiplash injuries is common and difficult to treat. In contrast impingement syndrome can be helped with physiotherapy, injection of corticosteroids and even surgery. It is therefore important for clinicians to suspect and correctly diagnose subacromial impingement in patients complaining of shoulder pain following neck injuries instead of merely blaming radicular neck pain as the cause. In fact careful assessment can even identify and successfully treat a group of patients who may present with 'asymptomatic impingement' with pain outside the neck and at the medial aspect of the scapula but not in the shoulder itself [12].

In our series all the patients with subacromial impingement had consulted their family doctor but only 9% had been referred to a specialist and less than a third had had their diagnosis made prior to our medicolegal report. None were diagnosed by their general practitioners. This study highlights the fact that a potentially treatable condition in a small group of patients is diagnosed late or not at all due to lack of awareness of the association between neck injury and subacromial impingement.

The exact cause of impingement syndrome associated with whiplash injuries is subject to debate. In our study the seatbelt shoulder was involved in 83% of cases (X^2^, P = 0.021) suggesting direct trauma from the seatbelt as a possible cause. Moreover all 11 patients had developed their symptoms early and between 1 and 7 days after the injury further supporting direct trauma as an underlying cause. Only two (17%) patients had symptoms in the non-seatbelt shoulder. But even these patients were found to have evidence of direct trauma to the non-seatbelt side of their body. 'Patient 4' who was a driver with left subacromial impingement was noted to have 'bruising' around the left elbow and forearm on the day of the accident. 'Patient 9' who was a driver with bilateral impingement (left worse than right) also had severe bruising and tenderness on the left chest wall and axilla after the accident and was admitted to hospital for analgesia and observation. In our study therefore, all of the shoulders that had developed subacromial impingement had been subject to direct trauma, by the seatbelt or otherwise.

The average age in the group of patients who developed subacromial impingement was higher than those without subacromial impingement: 57.5 years versus 36.9 years. This difference is statistically significant (T-test, p = 0.002). This suggests that age or pre-existing degenerative change leading to a decrease in the subacromial space may be a risk factor for developing subacromial impingement following direct trauma to the shoulder.

This study has several limitations. It is based on patients in legal proceedings and may not truly reflect the general population. The diagnosis of subacromial impingement was made on clinical grounds only and although imaging was available in a number of cases (table 1) it was not used universally. Injection of local anaesthetic into the subacromial space would have been a useful adjunct to the assessment of the cohort.

Although a significant number of seat-belted shoulders were identified, the numbers involved were small and a larger study needs to be conducted to confidently link seatbelt trauma to the development of impingement syndrome.

## Conclusion

Recent studies have suggested an association between whiplash injuries to the neck and shoulder pathology [6,12]. It has even been suggested that impingement syndrome can present without shoulder symptoms and with radicular neck pain alone [7]. This article is further validation that neck injury and impingement syndrome are associated. The exact incidence is unclear, however the diagnosis is commonly delayed due to lack of awareness of the potential association between whiplash and subacromial impingement and the assumption that all shoulder symptoms emanate from the neck.

Following a neck injury therefore, patients who present with pain outside the neck and radiating to the shoulder should be carefully assessed for evidence of subacromial impingement to avoid delay in the diagnosis of a potentially treatable condition.

## Authors' contributions

AA performed the data collection, the literature review and wrote the manuscript, GEG performed the medico-legal reporting, oversaw the data collection and helped in manuscript preparation.
